# Combined Tractional and Rhegmatogenous Retinal Detachment in Proliferative Diabetic Retinopathy in the Anti-VEGF Era

**DOI:** 10.1155/2014/917375

**Published:** 2014-06-25

**Authors:** Ya-Jui Hsu, Yi-Ting Hsieh, Po-Ting Yeh, Jehn-Yu Huang, Chung-May Yang

**Affiliations:** Department of Ophthalmology, National Taiwan University Hospital and Medical College, National Taiwan University, No. 7, Chung-Shan South Road, Taipei 100, Taiwan

## Abstract

*Purpose*. To investigate the clinical features, surgical outcomes, and prognostic factors of combined rhegmatogenous and tractional detachment (combined RD) in proliferative diabetic retinopathy (PDR) in recent years. *Methods*. Medical records of PDR and combined RD treated with vitrectomy from 2008 to 2013 were retrospectively reviewed. *Results*. A total of 57 eyes from 49 patients were included. Nine eyes had received panretinal photocoagulation (PRP) and 7 eyes had intravitreal bevacizumab (IVB) within 3 months before RD developed. Thirty-eight eyes (66.7%) had ≧3 sites of broad adhesion of fibrovascular proliferation (FVP). Thirty-three eyes (57.9%) showed active FVP. Thirty-four eyes (59.6%) had extent of RD involving 3 or 4 quadrants. The primary reattachment rate was 93.0%, and the final visual acuity (VA) improved by more than 3 lines in 80.7% of eyes. Neovascular glaucoma occurred in 4 eyes postoperatively. Poor preoperative VA, severe vitreoretinal adhesion, and broad extent of RD had significant correlation with poor visual outcomes. *Conclusion*. PRP or IVB might play a role in provoking combined RD. The anatomical and functional success rates of surgery were high. Poor preoperative VA and severe proliferations predicted poor visual outcomes.

## 1. Introduction

Combined tractional and rhegmatogenous retinal detachment (combined RD) is a rare but serious complication in proliferative diabetic retinopathy (PDR). A literature review shows that 7–35% of cases undergoing pars plana vitrectomy (PPV) for complications of diabetic retinopathy had combined RD [1–3], and the occurrence rate seems to have declined gradually over the past decades. The disease is characterized by the existence of retinal break(s) in association with fibrovascular proliferation (FVP) of various severities. The break(s) were mostly slit-like, round, or oval. They may be hidden within the retinal folds or covered by the adjacent FVP tissue. The detachment has a convex configuration and usually reaches the ora serrata in at least some areas, but the extension may be limited by panretinal photocoagulation (PRP) scars. We have documented the common clinical findings and surgical results of this complication before, [4] in the period prior to the popular use of antivascular endothelial growth factor (VEGF) agents (anti-VEGFs) for treating diabetic retinopathy.

As the use of anti-VEGFs became more common, alone or in combination with other agents, in treating PDR complications, clinical features and surgical success rates were likely altered. Anti-VEGFs may induce regression of active neovascularization and prevent the development of neovascular glaucoma (NVG); on the other hand, they may induce fibrous contractions, thus possibly causing more tractional RD (TRD) or combined RD. In this study, a retrospective analysis of consecutive patients with combined RD in PDR was performed to investigate clinical features, risk factors, and anatomical or functional success rates in recent years to assess the possible effect of anti-VEGFs on this specific PDR complication.

## 2. Materials and Methods

From January 2007 to January 2013, consecutive cases with combined RD treated by a single surgeon in National Taiwan University Hospital were retrospectively reviewed. The selection criterion for combined RD was convex-shaped detachment with varying severities of preretinal fibrovascular tissue. Exclusion criteria included cases with a macular hole, cases that were found to have combined RD after previous vitrectomy, and cases with follow-up times within 3 months after surgery. This study was approved by the ethical committee of the National Taiwan University Hospital.

Preoperative data, including the patient's age, sex, and systemic comorbidities, including hypertension, cardiovascular diseases, and renal diseases, were collected. A history of PRP or IVB within 3 months before the development of combined RD was also recorded. Preoperative ophthalmologic evaluations included measurements of best corrected visual acuity (BCVA) and intraocular pressure, biomicroscopic examination of the anterior and posterior segments, and fundus examination with indirect ophthalmoscopy. Abnormal changes such as cataract and iris rubeosis were also recorded. Funduscopic findings, including the presence of vitreous hemorrhage (VH), the size, shape, and number of retinal breaks, the extent of RD, the location and extension of preretinal fibrovascular proliferative tissue, and the proliferative features (see the last paragraph in the surgical technique section for definition), were also recorded.

### 2.1. Surgical Techniques

All patients underwent a standard 20- or 23-gauge 3-port PPV. Cases with active FVP (see the last paragraph in the surgical technique section for definition) were treated with IVB of 1.25 mg within 3 days before surgery. After core vitrectomy to remove opacified vitreous, an opening was created in the posterior hyaloid at a place where the retina was at a safe distance from the vitreous cortex. Anterior-posterior vitreoretinal traction was released as much as possible. The thick fibrotic plaque from the retina was then divided using scissors segmentation and delamination. Forceps were used to gently lift the fibrovascular tissue from the disc to release traction and to facilitate the identification of the surgical plane. Hyaloid removal toward the periphery was performed to the greatest possible extent. Thin and nontaut peripheral hyaloid still adherent to the retina was left unpeeled for fear of creating breaks. A vascular preretinal membranes were removed with forceps. In cases with severe FVP (Grade 4; see the last paragraph in the surgical technique section for definition) or large breaks, a bimanual technique was employed by using forceps in one hand and cutters or scissors in the other hand under chandelier light illumination. After complete release of all the traction force, air-fluid exchange was performed, followed by laser PRP around breaks and across the peripheral retina; C3F8 was then injected into the vitreous cavity for retinal tamponade. Silicone oil was used in eyes presenting with multiple large retinal breaks (preexisting or iatrogenic) or possible unfound breaks, eyes undergoing retinotomy or retinectomy, or those with residual vitreoretinal traction. A polytetrafluoroethylene (Gor-tex) encircling band with a width of 5 mm was placed between the ora serrata and the equator in the more severe cases. IVB was used at the end of surgery in every case. To reduce the possibility of over dosage in a gas or silicone oil filled eye, IVB of 0.75 mg (0.03 mL) was given. For cases given silicone oil tamponade, IVB was done before silicone oil infusion; for cases given gas tamponade, IVB was done after gas infusion. All patients were given topical antibiotics (3% gentamycin), steroids (1% prednisolone acetate), and mydriatics (1% atropine) after surgery for about 1 month and were regularly followed at the outpatient clinic for at least 3 months.

The records of intraoperative findings focused on the identification and confirmation of the number of retinal breaks, the extent of RD, and the extent of FVP. Certain risk factors were graded or defined to study their correlations with occurrence of TRD or visual prognosis. The extent of FVP was separated into 4 grades based on the severity of vitreoretinal adhesion: multiple-point adhesions with or without 1-site plaque-like broad adhesion (Grade 1), broad adhesions in more than 1 but fewer than 3 sites, located posterior to the equator (Grade 2), broad adhesions in more than 3 sites, located posterior to the equator or extending beyond the equator within 2 quadrants (Grade 3), and broad adhesions extending beyond more than 1 site anterior to equator (Grade 4). The extent of RD was recorded based on the number of detached quadrants. The proliferative features of affected eyes were separated into 2 types according to the vascular component of the proliferative tissue: the chronic fibrotic type, in which most of the proliferative tissue appeared avascular, and the active fibrovascular type, in which a larger part of the proliferative tissue contained visible vessels, accompanied by any degree of VH. Other findings such as intraocular pressure and the appearance of the retina and the disc at the last follow-up were recorded.

### 2.2. Statistical Analysis

Logarithm of the minimal angle of resolution (LogMAR) of BCVA was calculated, and we defined visual acuity of “counting fingers” level as LogMAR 2.3, “hand motion” level as LogMAR 2.7, and “light perception” as LogMAR 3. Linear mixed models were used for comparison between preoperative and postoperative LogMAR of BCVA with the patient as a random effect in order to capture the correlations between two eyes of the same patient. To evaluate the predictive factors for postoperative vision, linear mixed models were also used and the candidate factors included age, sex, preoperative LogMAR of BCVA, preoperative PRP, preoperative IVB, severity of vitreoretinal adhesion, the extent of RD, the vascular component of the proliferative tissue, retinal breaks found preoperatively or not, the number of retinal breaks found during operation, intraoperative retinectomy, silicone oil use, combined scleral buckling, combined cataract surgery, and recurrent RD with secondary PPV. Among these candidate factors, age and preoperative LogMAR of BCVA were treated as continuous variables. Sex, preoperative PRP, preoperative IVB, severity of vitreoretinal adhesion (Grade ≤ 3 versus Grade 4), the vascular component of the proliferative tissue, retinal breaks found preoperatively or not, the number of retinal breaks found during operation (single break versus multiple breaks), intraoperative retinectomy, silicone oil use, combined scleral buckling, combined cataract surgery, and recurrent RD with secondary PPV were treated as categorical variables. As to the extent of RD, the linear trend was evaluated. A *P* value less than 0.05 was considered statistically significant. SAS 9.1 (SAS Institute Inc., Cary, NC, USA) was used for all statistical analyses.

## 3. Results

Fifty-seven eyes of 49 patients with combined RD were enrolled in this study. The patients' characteristics are shown in Table 1. The mean age of the patients was 52.4 ± 9.4 years (range: 29 to 74 years), and 28 cases (57.1%) were female. The mean follow-up period was 24.4 ± 15.4 months (range: 3 to 72 months). In addition to diabetes, 34 cases (69.4%) had hypertension, 5 (10.2%) had coronary artery disease, 3 (6.1%) had stroke history, and 4 (8.2%) had chronic kidney disease.

Combined RD developed within 3 months following PRP in 7 cases (15.8%), within 3 months following IVB in 5 cases (8.8%), and within 3 months following PRP combined with IVB in another 2 cases (3.5%).

**Figure 1:**
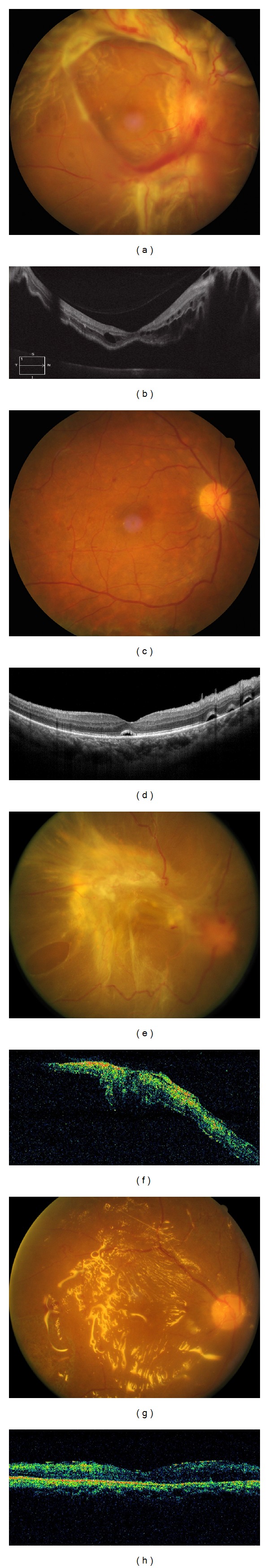
(a–d) Case 47 (right eye). (a) Preoperative fundus photo showed active fibrovascular proliferation of Grade 3 severity; (b) preoperative optical coherence tomography showed detached retina; (c) postoperative fundus photo showed retinal attachment 2 months after surgery; (d) postoperative OCT revealed flat central retina with residual subretinal fluid pockets. (e–h) Case 19 (right eye). (e) Preoperative fundus photo showed chronic fibrosis of Grade 3 severity. A large oval-shaped retinal break at temporal side was noted; (f) preoperative OCT showed elevated retina at the macular area; (g) postoperative fundus photo showed retinal attachment with silicone oil tamponade 1 month after surgery; (h) postoperative OCT revealed attached retina with thin epimacular membrane.

The clinical features of combined RD are shown in Table 2. The exact duration of RD could not be obtained with certainty since some patients had an RD diagnosis on their first visit to the outpatient clinic. Active neovascular RD was found in 57.9% of eyes (Figure 1(a)) and chronic fibrotic RD in 42.1% of eyes (Figure 1(e)). Only 27 eyes (47.4%) had breaks identified before surgery. The breaks were mostly oval- or slit-shaped. Thirty eyes (52.6%) had more than 1 break identified, and only 4 eyes had multiple breaks (>5) at the periphery. Severe (Grade 3 and Grade 4) FVP was noted in 45.6% and 21.1% of cases, respectively. Extension of RD ≧ 3 quadrants was found in more than 60% of eyes. Three eyes (5.3%) received retinectomy intraoperatively to release severe traction from the proliferative tissue. Silicone oil was used during primary PPV in 31 eyes (54.4%).

Overall, 53 eyes (93.0%) achieved primary retinal reattachment, and the remaining 4 eyes had final retinal reattachment after 1 or 2 more surgeries. Examples of preoperative and postoperative fundus pictures are shown in Figure 1.

After operation, the mean LogMAR of BCVA improved from 1.92 ± 0.68 to 1.22 ± 0.68 (*P* < 0.0001). Vision in forty-six eyes (80.7%) improved by 3 lines or more, while vision in 4 eyes (7.0%) deteriorated by 3 lines or more. Forty eyes (70.2%) maintained their BCVA≧20/400. However, one eye had no light perception postoperatively because of the development of NVG at 5 months after primary PPV and silicone oil tamponade.

Three cases had recurrent RD after primary PPV with C3F8 tamponade; all of them achieved reattachment after further PPV with silicone oil tamponade. One case (case 7) had recurrent RD after primary PPV with silicone oil infusion; this case received another PPV with removal and reinjection of silicone oil later and the retina was reattached. Other common postoperative complications included NVG in 4 eyes (7.0%), epiretinal membrane formation in 4 eyes (7.0%), foveal atrophy in 2 eyes (3.5%), recurrent VH in 2 eyes (3.5%), and retinal artery occlusion in 2 eyes (3.5%). Three of the 4 NVG cases had received PPV and silicone oil tamponade, with one case turning out to have no light perception. Only one case having received PPV and gas tamponade developed NVG later.

### 3.1. Predictive Factors for Postoperative BCVA

Table 3 shows the results of the predictive factors for postoperative BCVA. The preoperative BCVA was positively correlated with the postoperative BCVA (*P* = 0.002). Grade 4 vitreoretinal adhesion, intraoperative retinectomy, and silicone oil use were all correlated with a poorer postoperative BCVA (*P* = 0.01, 0.03, 0.02, and 0.01, resp.). The extent of RD also showed a linear trend of correlation with a poorer postoperative BCVA (*P* = 0.002). After adjustment for age, sex, and preoperative BCVA, Grade 4 vitreoretinal adhesion, intraoperative retinectomy, and a broader extent of RD were still correlated with a poorer postoperative BCVA (*P* = 0.04, 0.04, and 0.03, resp.).

## 4. Discussion

Combined RD is a serious PDR complication. Over the years, the occurrence rate of this disease entity has fluctuated. The ratio of combined RD to TRD was down from 1 : 2 in the 1980s to 1 : 6 in the 1990s [1]. More aggressive treatment of PDR in the early stages may contribute to the decline of combined RD. The use of intravitreal anti-VEGFs in recent years has engendered a dramatic change in treatment strategies for diabetic retinopathy. IVB has been widely used for diabetic macular edema, for NV with vitreous hemorrhage, and for facilitating surgery of active PDR in the preoperative period. Because IVB not only promotes NV regression, but also enhances fibrosis and contraction of the FVP tissue, it may facilitate the development of retinal breaks and thus alter the risk factors, clinical features, and treatment results of combined RD.

It has been shown that the effect of IVB may last for 4 to 6 weeks [5–7]. We arbitrarily set 3 months as the maximal time limit under which the retina might be influenced by the drug-induced FVP contraction. Previous studies have shown that the progression to TRD from a preexisting fibrovascular tissue contraction is a possible complication of IVB treatment [7–15]. In the present study, 5 cases had a history of IVB within 3 months before the development of combined RD, another 2 cases having both PRP and IVB. The severity of FVP in these 7 cases ranged from Grades 2 to 3. Thus it appears that retinal breaks might occur in FVP of various severities. PRP as a risk factor for combined RD has not been reported [4]. In this study, 7 cases had a history of PRP within 3 months before the development of combined detachment, and another 2 cases had a history of both PRP and IVB. The laser energy itself and the laser-induced inflammation may cause vitreous contraction. Active FVP may also cause progressive retinal traction. These forces may pull on the laser treated area and induce retinal breaks from the recently applied laser spots, resulting in combined RD. Furthermore, our observations suggested that combined RD may develop in an eye already reaching a quiescent or even burnout stage with various degrees of preretinal fibrosis. Thus, although lacking definite statistical support, the percentage of recent PRP and/or IVB in cases with combined RD (more than 10%) suggested that one should perform these procedures with caution, as these procedures may possibly predispose eyes to increased traction or even combined RD.

In this study, an anti-VEGF (bevacizumab) was used preoperatively only in those cases with severe active proliferation, and operation was done within 3 days after injection to decrease the possibility of more vitreous traction. The benefit of IVB at the end of operation has been debated [16–19]. Previous studies also have shown that vitreous cavity tamponade by long-acting gas or silicone oil reduced the incidence of recurrent VH [20, 21]. Although the rate of recurrent VH in the present study was low, the benefit of IVB on postoperative recurrent VH was inconclusive due to the lack of a control group.

Based on the risk factors and clinical manifestations, we suggest the following possible pathways for the development of combined RD: first, active FVP with TRD undergoes further traction to induce break formation; second, the fibrotic tissue and the vitreous exert traction on the atrophic retina in a chronic PDR with or without TRD, causing break formation; third, vitreous anterior-posterior directed traction on the adherent FVP tissue induces flap tear formation; fourth, traction induced by PRP or anti-VEGF on attached or detached retina causes formation of breaks.

The significant prognostic factors in the present study were preoperative BCVA, vitreoretinal adhesion, extent of RD, intraoperative retinectomy, and silicone oil use, all of which might be interrelated. A larger extent of RD is usually accompanied by a severe vitreoretinal adhesion in severe PDR cases. Poor preoperative BCVA may be associated with active fibrovascular RD and dense VH or extensive RD with macular involvement. In addition, intraoperative retinectomy and silicone oil use were often applied in cases with severe proliferation and a large area of RD.

Clinical pictures and treatment results of combined RD have been reported previously [4].  Table 4 offers a comparison of the clinical manifestations between the present study and the previous one. Both studies showed similar primary reattachment rates (over 90% in both) and postoperative VA improvement rates (81% in the present study versus 70% in the previous one). However, some differences between these two studies were found. In the present one, there were more cases with more than one break (52.6% versus 30%, *P* = 0.03) or with breaks identified before surgery (47.4% versus 17.5%, *P* = 0.002). The ratio of active type versus chronic type of PDR was 1.38 in the present study, as opposed to 0.90 in the previous one. Although there were slight differences in the grading systems employed in the two studies, severe FVP (Grades 3 and 4) was noted in 66.7% of cases in the present study, as compared to 52.5% in the previous one. Total RD was noted in 33.3% of cases in the present study but reached 55% in the previous one. It is possible that the frequent use of IVB may be associated with increased break formation in the active phase of PDR, causing the differences between the two studies. The less extent of RD may be partly explained by the more aggressive PRP treatment in recent years, since VH has quicker clear-up rate after IVB, and laser spots can more easily be applied to the retina.

A higher proportion of cases with better postoperative vision (BCVA ≥ 0.05) were noted in the present study than in the previous one (70.2% versus 47.5%, *P* = 0.02); this difference might be partly due to fewer cases of chronic fibrosis type in the present study and the use of adjunct therapy of IVB before surgery in the active cases. However, NVG developed in 7.0% of cases in the present study, which was similar to the previous one. This means that the use of IVB as a surgical adjunct at the end of operation may not be able to eliminate the development of NVG. As IVB only has temporary effects, careful monitoring of the presence of rubeosis and IOP are mandatory to decrease the occurrence of this disastrous complication.

In this study, the prognostic factors were somewhat different from our previous study. We enrolled more candidate prognostic factors for analysis in the present study. Placing all candidate factors into the same regression model would result in collinearity, since many of them were related to the severity of diseases. Therefore, only age, sex, and preoperative BCVA were adjusted for when evaluating these prognostic factors in linear mixed models, under the belief that this could more accurately unveil the relationships between visual outcomes and these candidate prognostic factors.

In this retrospective study, case recruitment bias and uneven follow-up intervals and durations of study patients were inevitable. Our hospital is a tertiary referral medical center, PRP or IVB had been done in some cases at original hospitals, and the timing of treatment might be different. The severities of the fundus changes of individual case were not clear before PRP/IVB treatment. However, the case number was relatively large; a single experienced surgeon operated on all case patients and precise chart recordings were maintained throughout follow-ups. Our study found that, with modern instruments and techniques, the anatomical success rate was high, and visual outcome usually showed significant improvement. The clinical features of combined RD in recent years seem to differ in several aspects from those in the period when anti-VEGFs were not popularly used. More active cases and more severe FVP were noted in the present study than the previous study. The rate of preoperative break identification was also higher. Poorer preoperative VA, intraoperative retinectomy, and higher grading of FVP are associated with poorer postoperative visual improvement. Previous PRP or IVB may possibly play a role in provoking combined RD. The use of anti-VEGFs may not prevent the complication of NVG.

## Figures and Tables

**Table 1:** Clinical characteristics of enrolled patients.

Case	Age (years)	Sex	Left/right eye	Comorbidities	Preop. PRP	Preop. IVB	Surgery	F/U period (months)	Anatomic outcome
1	54	F	L	HTN	+	−	PPV + SB + SO	13	Attached
2	47	F	R	HTN	−	+	PPV + SO	13	Attached
3	53	F	L	—	−	−	Phaco + PPV + SB + SO	22	Attached
4	43	M	R	HTN	−	−	Phaco + PPV + SB + C3F8	60	Recurrent RD
5	48	M	L	CAD	−	−	PPV + SB + SO	46	Attached
6	51	M	L	—	−	−	PPV + SB + SO	17	Attached
			R		−	−	PPV + SB + SO	15	Attached
7	62	M	L	HTN	−	−	PPV + SB + SO	32	Recurrent RD
8	61	M	R	HTN	−	−	Phaco + PPV + C3F8	17	Attached
9	52	F	L	HTN	−	−	PPV + SB + SO	14	Attached
10	63	F	R	HTN	−	−	PPV + C3F8	35	Attached
11	54	M	R	HTN, stroke	−	−	PPV + SB + C3F8	40	Attached
12	54	M	L	HTN, CAD	−	−	Phaco + PPV + SB + SO	60	Attached
13	60	F	L	HTN, CAD	−	−	Phaco + PPV + SB + SO	11	Attached
14	68	F	R	HTN, stroke	−	−	PPV + SB + C3F8	35	Attached
15	62	F	R	—	−	−	Phaco + PPV + SO	8	Attached
16	56	F	L	HTN	−	−	Phaco + PPV + SB + SO	27	Attached
17	51	F	L	HTN	−	−	PPV + SB + C3F8	55	Recurrent RD
			R		−	−	PPV + C3F8	32	Attached
18	33	M	R	HTN	+	−	PPV + SB + SO	43	Attached
19	52	F	R	HTN	+	+	PPV + SO	26	Attached
			L		−	−	PPV + SO	9	Attached
20	29	M	R	HTN	−	−	PPV + C3F8	8	Attached
21	52	F	R	HTN	−	+	PPV + SB + C3F8	61	Attached
22	56	F	L	HTN	−	+	PPV + SB + C3F8	5	Attached
23	52	F	L	HTN, CKD	−	−	PPV + SB + SO	31	Attached
24	47	F	R	HTN	−	−	Phaco + PPV + SB + Retinectomy + SO	22	Attached
25	55	M	L	—	−	−	PPV + C3F8	34	Attached
26	55	F	R	HTN	−	−	PPV + C3F8	26	Attached
			L		+	−	PPV + SB + SO	12	Attached
27	64	F	R	HTN	−	−	PPV + SB + C3F8	48	Attached
28	63	M	R	HTN	−	−	Phaco + PPV + SB + Retinectomy + SO	5	Attached
29	57	F	R	—	−	−	PPV + Retinectomy + SO	17	Attached
30	65	M	R	—	−	−	PPV + C3F8	48	Attached
31	30	F	L	HTN	−	−	PPV + SB + SO	10	Attached
32	54	F	L	—	−	−	PPLV + SB + SO	22	Attached
33	37	M	R	HTN	+	−	PPV + C3F8	45	Recurrent RD
			L		−	+	PPV + SB + SO	40	Attached
34	50	M	L	HTN, CKD	−	−	Phaco + PPV + SB + SO	6	Attached
35	47	F	L	HTN, CAD, CKD	−	−	PPV + C3F8	72	Attached
			R		−	−	PPV + C3F8	70	Attached
36	74	M	L	HTN, stroke, CAD	−	−	PPV + SB + C3F8	3	Attached
37	38	F	L	HTN	−	−	PPV + SB + C3F8	19	Attached
38	47	F	R	—	−	−	PPV + SB + SO	5	Attached
39	50	M	R	—	−	−	PPV + SB + C3F8	6	Attached
40	47	M	R	—	−	−	PPV + C3F8	15	Attached
41	41	F	L	—	−	−	Phaco + PPV + SB + SO	26	Attached
42	50	M	L	HTN	+	−	PPV + SB + SO	17	Attached
43	51	M	L	—	+	−	PPV + SB + C3F8	31	Attached
44	45	M	R	HTN, CKD	−	−	PPV + SB + SO	6	Attached
45	67	M	L	HTN	+	−	Phaco + PPV + C3F8	8	Attached
			R		−	−	PPV + C3F8	6	Attached
46	60	F	R	HTN	−	−	PPV + SB + SO	6	Attached
			L		−	−	PPV + SB + SO	6	Attached
47	53	F	R	—	−	+	PPV + C3F8	8	Attached
48	54	F	R	HTN	−	−	PPV + SO	8	Attached
49	56	F	R	—	+	+	PPV + SB + SO	6	Attached

CAD: coronary artery disease; CKD: chronic kidney disease; F: female; HTN: hypertension; M: male; phaco: phacoemulsification; PPV: pars plana vitrectomy; RD: retinal detachment; SB: scleral buckling; SO: silicone oil.

**Table 2:** Clinical features and outcome of combined retinal detachment.

Case	Left/right eye	Extent of RD (quadrant)	Severity of vitreoretinal adhesion (grade)	Vascular component of proliferative tissue (chronic fibrotic versus active fibrovascular proliferative)	Retinal breaks found preop. or not	Number of retinal breaks (single versus multiple breaks)	Preop. VA (logMAR)	Post-op. VA (logMAR)	Complication
1	L	3	3	Chronic	−	Multiple	2	1.5	—
2	R	2	2	Active	−	Multiple	1.7	0.7	RAO
3	L	4	4	Active	−	Multiple	HM/20 cm	CF/20 m	Foveal atrophy
4	R	3	1	Active	+	Multiple	HM/1 m	1.7	—
5	L	3	3	Chronic	+	Multiple	2	0.7	—
6	L	2	3	Active	−	Multiple	2	1	—
	R	4	4	Chronic	+	Multiple	HM/1 m	NLP	NVG
7	L	3	2	Chronic	+	Multiple	CF/70 cm	1	—
8	R	1	1	Chronic	+	Multiple	1	0.3	Recurrent VH
9	L	2	4	Chronic	+	Multiple	CF/30 cm	1.3	—
10	R	1	1	Active	−	Single	0.7	0.22	—
11	R	2	2	Active	+	Multiple	1.5	0.52	NVG
12	L	3	2	Chronic	+	Single	1.3	1.4	—
13	L	4	4	Chronic	−	Single	1.3	0.82	—
14	R	3	3	Active	−	Single	1.3	1	—
15	R	2	4	Chronic	−	Single	HM/30 m	1.52	—
16	L	3	4	Chronic	+	Single	HM/1 m	0.7	ERM
17	L	3	4	Chronic	−	Multiple	0.52	1.3	Foveal atrophy
	R	1	1	Chronic	+	Single	1.7	1	—
18	R	3	3	Active	+	Single	1.7	0.52	—
19	R	4	3	Chronic	+	Single	CF/30 cm	1.3	—
	L	4	3	Active	−	Multiple	HM/50 cm	HM/1 m	—
20	R	4	3	Active	−	Single	CF/20 cm	0.7	—
21	R	2	3	Chronic	−	Multiple	0.7	0.82	—
22	L	2	2	Active	+	Single	0.82	0.52	Recurrent VH, RAO
23	L	3	3	Active	−	Single	2	1	ERM
24	R	2	4	Active	−	Multiple	2	1.7	—
25	L	4	3	Active	−	Multiple	HM/30 cm	0.7	—
26	R	1	3	Active	+	Multiple	0.52	0.22	—
	L	3	3	Active	+	Single	1.7	0.82	NVG
27	R	2	2	Active	−	Multiple	2	1	—
28	R	4	3	Active	−	Single	CF/60 cm	CF/60 cm	—
29	R	4	4	Active	−	Single	2	HM/40 cm	NVG
30	R	2	2	Chronic	−	Single	2	1.3	—
31	L	4	3	Active	+	Single	HM/1 m	1.3	—
32	L	4	4	Active	+	Multiple	HM/10 cm	HM/30 cm	—
33	R	3	3	Active	+	Single	1.7	1.3	—
	L	3	3	Active	−	Multiple	CF/50 cm	1	—
34	L	4	4	Chronic	−	Multiple	CF/30 cm	1.3	—
35	L	2	2	Active	−	Single	2	0.7	—
	R	2	2	Active	−	Multiple	0.4	0.82	ERM
36	L	2	2	Active	+	Multiple	HM/1 m	1.7	—
37	L	4	1	Chronic	−	Multiple	1.7	1	—
38	R	3	4	Active	−	Multiple	1.7	1.52	—
39	R	1	2	Active	−	Multiple	1.52	1.7	—
40	R	2	2	Active	+	Multiple	CF/50 cm	0.52	—
41	L	4	1	Chronic	+	Single	HM/50 m	CF/60 cm	—
42	L	4	2	Chronic	+	Multiple	CF/20 cm	1.7	—
43	L	4	3	Active	−	Single	HM/1 m	0.82	—
44	R	3	3	Chronic	+	Multiple	HM/60 cm	1.3	—
45	L	2	3	Chronic	−	Single	1.5	0.82	ERM
	R	1	3	Active	−	Single	1.3	0.7	—
46	R	4	3	Chronic	+	Single	1.7	1.3	—
	L	4	3	Chronic	+	Single	1.3	0.7	—
47	R	2	3	Active	−	Single	0.7	0.22	—
48	R	4	3	Active	+	Multiple	HM/1 m	CF/40 cm	—
49	R	2	2	Chronic	+	Single	1.6	0.82	—

CF: counting fingers; ERM: epiretinal membrane; HM: hand motion; NLP: nonlight perception; NVG: neovascular glaucoma; RAO: retinal artery occlusion; VH: vitreous hemorrhage.

The severity of vitreoretinal adhesion was defined as multiple-point adhesions with or without 1-site-wide plaque-like adhesion (Grade 1), broad adhesions in more than 1 but fewer than 3 sites posterior to equator (Grade 2), broad adhesions in more than 3 sites posterior to equator or 1 site anterior to equator (Grade 3), and broad adhesions more than 1 site anterior to equator (Grade 4).

**Table 3:** Regression analyses of risk factors associated with poor postoperative visual outcomes.

Predictive factors	Simple regression		Adjustment for age, sex, and preoperative BCVA	
	Coefficient	*P* value	Coefficient	*P* value
Age	0.0003	0.98	—	—
Sex (F versus M)	−0.085	0.66	—	—
Preoperative BCVA	0.558	0.002∗	—	—
Preoperative PRP	0.062	0.77	−0.017	0.92
Preoperative IVB	−0.085	0.09	0.016	0.93
Severity of vitreoretinal adhesion (Grade ≤ 3 versus Grade 4)	0.697	0.01∗	0.502	0.04∗
Vascular component of proliferative tissue (chronic fibrotic versus active fibrovascular)	−0.050	0.80	−0.008	0.96
Extent of RD	0.358	0.002∗	0.248	0.03∗
Retinal breaks found preoperatively or not	0.059	0.76	−0.074	0.66
Number of retinal breaks (single break versus multiple breaks)	0.251	0.21	0.242	0.17
Intraoperative retinectomy	0.854	0.02∗	0.681	0.04∗
Silicone oil use	0.593	0.01∗	0.326	0.12
Combined scleral buckling	0.301	0.16	0.188	0.30
Combined cataract surgery	0.199	0.34	0.197	0.28
Recurrent RD with secondary PPV	0.334	0.33	0.357	0.24

BCVA: best corrected visual acuity; IVB: intravitreal bevacizumab; RD: retinal detachment; PRP: panretinal photocoagulation.

*P < 0.05 was considered to be statistically significant.

**Table 4:** Comparison of the present study and the previous study.

	Current study		Yang et al., 2008 [4]		*P* value
Eyes (patients)	57 (49)		40 (36)		—
Age (years) [mean ± SD (range)]	52.4 ± 9.4 (29–74)		54.7 ± 11.3 (25–70)		0.14
Active : chronic (ratio)	33 : 24 (1.38)		19 : 21 (0.90)		0.31
Multiple breaks [*n* (%)]	30 (52.6)		12 (30)		0.03∗
Break(s) noted before surgery [*n* (%)]	27 (47.4)		7 (17.5)		0.002∗
Extent of FVP					0.35
Grade 1 [*n* (%)]	6 (10.5)		5 (12.5)		
Grade 2 [*n* (%)]	13 (22.8)		14 (35)		
Grades 3 and 4 [*n* (%)]	38 (66.7)		21 (52.5)		
Extent of RD					0.09
Total [*n* (%)]	19 (33.3)		22 (55)		
Subtotal [*n* (%)]	32 (56.1)		14 (35)		
Localized [*n* (%)]	6 (10.5)		4 (10)		
Primary retina reattachment [*n* (%)]	53 (93.0)		37 (92.5)		0.93
Post-op VA					
Improved	46 (80.7)	28 (70)	Improved	28 (70)	0.57
Unchanged	6 (10.5)	6 (15)	Unchanged	6 (15)	
Worse	5 (8.8)	6 (15)	Worse	6 (15)	
Post-op VA ≥ 0.05 [*n* (%)]	40 (70.2)		19 (47.5)		0.02∗
Post-op NVG [*n* (%)]	4 (7.0)		3 (7.5)		0.93

FVP: fibrovascular proliferation; OP: operation; RD: retinal detachment; VA: visual acuity. Statistical analyses of noncontinuous variables were performed with chi-squared test as appropriate, and continuous variables were performed with two-sample *t*-test. *P value of less than 0.05 was considered statistically significant.

## References

[1] Yang C-M (1998). Surgical treatment for diabetic retinopathy: 5-year experience. *Journal of the Formosan Medical Association*.

[2] Thompson JT, de Bustros S, Michels RG, Rice TA, Glaser BM (1986). Results of vitrectomy for proliferative diabetic retinopathy. *Ophthalmology*.

[3] Aaberg TM, Abrams GW (1987). Changing indications and techniques for vitrectomy in management of complications of diabetic retinopathy. *Ophthalmology*.

[4] Yang C-M, Su P-Y, Yeh P-T, Chen M-S (2008). Combined rhegmatogenous and traction retinal detachment in proliferative diabetic retinopathy: clinical manifestations and surgical outcome. *Canadian Journal of Ophthalmology*.

[5] Tonello M, Costa RA, Almeida FPP, Barbosa JC, Scott IU, Jorge R (2008). Panretinal photocoagulation versus PRP plus intravitreal bevacizumab for high-risk proliferative diabetic retinopathy (IBeHi study). *Acta Ophthalmologica*.

[6] Jorge R, Costa RA, Calucci D, Cintra LP, Scott IU (2006). Intravitreal bevacizumab (Avastin) for persistent new vessels in diabetic retinopathy (IBEPE study). *Retina*.

[7] Moradian S, Ahmadieh H, Malihi M, Soheilian M, Dehghan MH, Azarmina M (2008). Intravitreal bevacizumab in active progressive proliferative diabetic retinopathy. *Graefe’s Archive for Clinical and Experimental Ophthalmology*.

[8] Spaide RF, Fisher YL (2006). Intravitreal bevacizumab (Avastin) treatment of proliferative diabetic retinopathy complicated by vitreous hemorrhage. *Retina*.

[9] Arevalo JF, Wu L, Sanchez JG (2009). Intravitreal bevacizumab (avastin) for proliferative diabetic retinopathy: 6-Months follow-up. *Eye*.

[10] Huang Y-H, Yeh P-T, Chen M-S, Yang C-H, Yang C-M (2009). Intravitreal bevacizumab and panretinal photocoagulation for proliferative diabetic retinopathy associated with vitreous hemorrhage. *Retina*.

[11] Di Lauro R, De Ruggiero P, Di Lauro R, Di Lauro MT, Romano MR (2010). Intravitreal bevacizumab for surgical treatment of severe proliferative diabetic retinopathy. *Graefe’s Archive for Clinical and Experimental Ophthalmology*.

[12] Pokroy R, Desai UR, Du E, Li Y, Edwards P (2011). Bevacizumab prior to vitrectomy for diabetic traction retinal detachment. *Eye*.

[13] Oshima Y, Shima C, Wakabayashi T (2009). Microincision vitrectomy surgery and intravitreal bevacizumab as a surgical adjunct to treat diabetic traction retinal detachment. *Ophthalmology*.

[14] Arevalo JF, Maia M, Flynn HW (2008). Tractional retinal detachment following intravitreal bevacizumab (Avastin) in patients with severe proliferative diabetic retinopathy. *British Journal of Ophthalmology*.

[15] Jonas JB, Schmidbauer M, Rensch F (2009). Progression of tractional retinal detachment following intravitreal bevacizumab. *Acta Ophthalmologica*.

[16] Romano MR, Gibran SK, Marticorena J, Wong D, Heimann H (2009). Can an intraoperative bevacizumab injection prevent recurrent postvitrectomy diabetic vitreous hemorrhage?. *European Journal of Ophthalmology*.

[17] Cheema RA, Mushtaq J, Al-Khars W, Al-Askar E, Cheema MA (2010). Role of intravitreal bevacizumab (Avastin) injected at the end of diabetic vitrectomy in preventing postoperative recurrent vitreous hemorrhage. *Retina*.

[18] Park DH, Shin JP, Kim SY (2010). Intravitreal injection of bevacizumab and triamcinolone acetonide at the end of vitrectomy for diabetic vitreous hemorrhage: a comparative study. *Graefe’s Archive for Clinical and Experimental Ophthalmology*.

[19] Ahn J, Woo SJ, Chung H, Park KH (2011). The effect of adjunctive intravitreal bevacizumab for preventing postvitrectomy hemorrhage in proliferative diabetic retinopathy. *Ophthalmology*.

[20] Yang C-M, Yeh P-T, Yang C-H (2007). Intravitreal long-acting gas in the prevention of early postoperative vitreous hemorrhage in diabetic vitrectomy. *Ophthalmology*.

[21] Bodanowitz S, Kir N, Hesse L (1997). Silicone oil for recurrent vitreous hemorrhage in previously vitrectomized diabetic eyes. *Ophthalmologica*.

